# Long-Term Outcomes of Living Kidney Donors in a Developing Country: A Single-Center Study

**DOI:** 10.3390/jcm14248908

**Published:** 2025-12-17

**Authors:** Alparslan Güneş, Gizem Kumru, Ebru Dumlupınar, Şule Şengül, Kenan Keven

**Affiliations:** 1 Department of Nephrology, Faculty of Medicine, Ankara University, 0600 Ankara, Türkiyesule.sengul@medicine.ankara.edu.tr (Ş.Ş.);; 2 Department of Biostatistics, Faculty of Medicine, Ankara University, 0600 Ankara, Türkiye; eedumlupinar@gmail.com

**Keywords:** living kidney donor, kidney transplantation, chronic kidney disease, cardiovascular risk

## Abstract

**Background/Objectives**: Kidney transplantation remains the most effective treatment for patients with end-stage kidney disease, increasing both survival and quality of life. There are concerns regarding the long-term outcomes of donors in developing countries, as kidney transplants are predominantly performed from living donors. This study was conducted to evaluate the long-term clinical outcomes of living kidney donors, with a particular focus on kidney and cardiovascular health. **Methods**: We retrospectively reviewed the records of 232 individuals who underwent donor nephrectomy between January 2011 and November 2022. Cardiovascular events, mortality, chronic kidney disease, hypertension, and newly onset diabetes were assessed. Estimated glomerular filtration rate (eGFR) values were employed to monitor kidney function over time. **Results**: Living kidney donors were monitored for a median of 6 years (IQR: 4–9 years). During the follow-up period, 18.9% of donors experienced a decline in eGFR to below 60 mL/min/1.73 m^2^; however, none progressed to end-stage kidney disease. Of the cohort, 20 (8.6%) had newly onset proteinuria and none had proteinuria before transplantation. Although there were no recorded deaths from cardiovascular causes, 4.3% of donors experienced major adverse cardiac events. 12.3% of donors had newly diagnosed hypertension following transplantation, and 20.2% of donors had hypertension overall. Lower baseline eGFR, treated as a continuous variable in the logistic regression model, was independently associated with a higher likelihood of post-donation eGFR < 60 mL/min/1.73 m^2^ (OR: 0.91; 95% CI: 0.88–0.94; *p* < 0.001). Post donation proteinuria (OR: 6.61; 95% CI: 1.98–22.07, *p*: 0.002) was also identified as independent risk factors for decline in eGFR to below 60 mL/min/1.73 m^2^. Diabetes mellitus was found to be a significant predictor of newly onset hypertension. **Conclusions**: A considerable percentage of the donors experienced gradual deterioration in kidney function, even though none of them developed kidney failure necessitating dialysis. The prevalence of obesity and chronic kidney disease was higher post-donation compared to the general population, indicating the need for structured long-term monitoring.

## 1. Introduction

Living kidney donor transplantation is a preferred method for treating end-stage kidney disease (ESKD). Worldwide, 39% of the 111,135 kidney transplants were conducted using living donors; however, in developing nations, living donors have emerged as the primary source of transplantation. In Europe, kidney transplants from deceased donors constituted 66%, while those from living donors accounted for 33%; however, Türkiye demonstrated the highest prevalence, with over 90% of kidney transplants originating from living donors [1].

The elevated incidence of living donor kidney transplants in Türkiye is affected by religious, sociocultural, and healthcare system variables. Strong familial connections, the view of living kidney donation as an altruistic endeavor, and the predominantly favorable perspective on living donation within mainstream religion may account for this elevated rate. Moreover, the restricted availability of cadaveric kidneys further enhances the inclination towards living donors.

Multiple studies have investigated the clinical implications of decreased nephron mass subsequent to donor nephrectomy. Studies demonstrate that while long-term survival rates for kidney donors and non-donors are similar, donors may face a heightened risk of hypertension (HT), chronic kidney disease (CKD), and atherosclerotic cardiovascular disease compared to healthy non-donor counterparts [2,3,4,5,6]. Considering that living kidney donation constitutes the predominant practice in developing nations, it is essential to assess the long-term health of living donors and establish suitable interventions in these environments. This study intends to evaluate the cardiac and kidney outcomes of donors at our facility, as well as the related risk factors.

## 2. Materials and Methods

We evaluated the clinical and demographic information of 379 kidney donors who underwent surgery at our transplantation center from January 2011 to November 2022. We included a total of 232 donors who had a minimum follow-up duration of one year. The follow-up examinations at the outpatient clinic post-transplantation were evaluated. Laboratory data, including fasting plasma glucose, serum creatinine, estimated glomerular filtration rate (eGFR), and urine protein/creatinine ratio (UPCR), as well as medical data such as blood pressure, body mass index (BMI), comorbidities, and patient survival, were documented.

Hypertension is diagnosed when the diastolic blood pressure surpasses 90 mmHg, the systolic blood pressure exceeds 140 mmHg, and/or the patient is receiving prescribed antihypertensive medication. Indicators of diabetes mellitus include a fasting plasma glucose level exceeding 126 mg/dL, the administration of prescription antidiabetic medication, a HbA1c level greater than 6.5%, and the presence of a documented diabetes diagnosis in medical records. A patient was designated as having atherosclerotic cardiovascular disease (ASCVD) if this condition was recorded in their medical records and if they were prescribed medications specifically for it. Additionally, a BMI of 30 kg/m^2^ or higher was considered for the diagnosis of obesity. Risk factors associated with a reduction in eGFR below 60 mL/min/1.73 m^2^ were examined.

The ethical committee of Ankara University approved the study protocol (ID: 2022/608, date: 19 December 2022), and it was conducted in compliance with the Declaration of Helsinki. Informed consent was not acquired owing to the study’s retrospective design.

### 2.1. Donor Selection Process

All potential living kidney donors underwent a standardized, pre-donation evaluation in accordance with our institutional transplant unit protocol and national transplant regulations. The screening process began with a comprehensive medical history and detailed physical examination, with particular emphasis on prior kidney disease, cardiovascular risk factors, metabolic disorders, and family history of kidney and systemic diseases.

Blood pressure was assessed using standardized office-based techniques on several occasions. Baseline kidney function was evaluated using the creatinine clearance in 24-h urine at least two different measurements, serum creatinine-based estimated glomerular filtration rate (eGFR), calculated with the CKD-EPI formula. Urinary protein excretion was assessed through standard dipstick urinalysis and subsequently quantified via spot urine protein and albumin-to-creatinine ratio measurements to rule out clinically significant proteinuria.

All donors underwent kidney imaging with ultrasonography and contrast-enhanced computed tomography angiography to assess kidney parenchyma, vascular anatomy, and urological suitability for donation. A thorough laboratory assessment was conducted, including complete blood count, serum electrolytes, liver function tests, fasting plasma glucose, glycated hemoglobin as necessary, and lipid profile.

Cardiovascular risk assessment was based on medical history, physical examination, resting electrocardiography, and additional cardiological testing (such as echocardiography or stress testing) when clinically indicated.

Infectious disease screening was performed in accordance with national transplant regulations and included testing for hepatitis B and C viruses, human immunodeficiency virus, and other pathogens required by the region.

All donor candidates also underwent mandatory psychiatric and psychosocial evaluation by a psychiatrist and the transplant team to assess motivation, decision-making capacity, psychological suitability, and the absence of coercion. Only individuals who fulfilled all medical, surgical, and psychosocial eligibility criteria were approved for donation. Donors with uncontrolled hypertension, diabetes with end-organ involvement, significant proteinuria, or eGFR below accepted thresholds were excluded from the donation program.

### 2.2. Statistical Analysis

Descriptive statistics were presented as mean ± standard deviation for the variables distributed normally and as median (min, max, IQR) for the variables distributed not normally, whereas they were presented as number and percentage (%) for nominal variables. The significance of the difference between the groups in terms of the mean values was analyzed by Student’s *t*-test, and the significance of the difference between the groups in terms of the median values was analyzed by Mann–Whitney U test. Categorical variables were evaluated using Pearson’s chi-squared test or Fisher’s exact test as appropriate. To define independent risk factors of outcome variable (eGFR to below 60 mL/min/1.73 m^2^), univariate and multivariate logistic regression analysis was used. To avoid multicollinearity, the most clinically important risk factor among statistically highly correlated risk factors was included in the multiple logistic regression analysis. Adjusted odds ratios (OR) and their 95% confidence intervals were calculated. Multivariate analysis was performed using a logistic regression that included variables (*p*-value < 0.20) in the univariate analysis. In order to control for the potential effects of the patient’s gender and age, adjusted models were created in the multivariate logistic regression analyses. This approach allowed us to assess the effects of the primary independent variables more clearly and helped to eliminate the potential confounding factors from the results. A *p* value of less than 0.05 was considered statistically significant and the analyses were conducted using the Statistical Package for Social Sciences (SPSS, Version 30.0, Chicago, IL, USA).

## 3. Results

In a cohort of 232 donors, the average age at transplantation was 49.30 ± 10.46 years, with a predominance of females (62.1%). The average eGFR at the time of transplantation was 103.68 ± 10.70 mL/min/1.73 m^2^, comparable between both genders. (*p* = 0.134). Active smoking was more common in men than in women (40.9% vs. 11.8%, *p* < 0.001), while the prevalence of post-transplantation obesity was greater in women (39.2% vs. 21.5%, respectively). Ten donors exhibited atherosclerotic cardiovascular disease (ASCVD), while 21 donors had a history of hypertension prior to transplantation. Demographics of donor population are shown below (Table 1).

**Figure 1:**
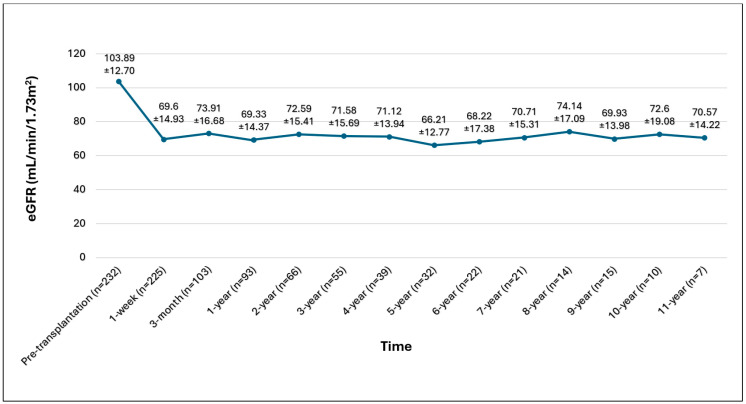
Graphical eGFR demonstration of overall kidney donors.

The average eGFR one-week post-donor nephrectomy was 69.60 ± 14.93 mL/min/1.73 m^2^, maintaining stability thereafter (Figure 1).

Figure 1. Graphical eGFR demonstration of overall kidney donors.

An examination of eGFR values following transplantation revealed that 44 donors (18.9%) exhibited a reduction in eGFR to 60 mL/min 1.73 m^2^ during the follow-up period, including two patients (0.86%) with eGFR levels between 30 and 45 mL/min/1.73 m^2^. All donors had an eGFR exceeding 30 mL/min/1.73 m^2^ and did not need kidney replacement therapy. The average age at transplantation was higher in donors with an eGFR below 60 mL/min/1.73 m^2^ post-transplantation compared to those above this threshold (54.7 ± 10.1 vs. 48.7 ± 10.4 years, *p* < 0.001) (Table 2).

Among the donors, 20 (8.6%) demonstrated a UPCR exceeding 150 mg/g post donation. Mean post donation UCPR was 110.67 ± 75.16 mg/g creatinine. Risk factors for a decline in eGFR below 60 mL/min/1.73 m^2^ included age over 50 at the time of donation (OR: 3.68; 95% CI: 1.78–7.59; *p* < 0.001), the presence of ASCVD (OR: 3.37; 95% CI: 1.20–9.42; *p* = 0.021), and newly onset proteinuria (OR: 4.14; 95% CI: 1.60–10.73; *p* = 0.003), all of which were significantly correlated with an elevated risk. The pre-transplant eGFR exhibited an inverse correlation with the reduction in eGFR below 60 mL/min/1.73 m^2^ (OR: 0.91; 95% CI: 0.88–0.94; *p* < 0.001). In multivariable analysis, proteinuria persisted as an independent risk factor for a decline in eGFR below 60 mL/min/1.73 m^2^ (OR: 6.61; 95% CI: 1.98–22.07; *p* = 0.002), whereas a higher pre-transplant eGFR continued to exhibit a protective effect (OR: 0.91; 95% CI: 0.88–0.95; *p* < 0.001) (Table 3).

Post-transplantation, it was observed that 7 donors (3.01%) experienced newly onset diabetes mellitus. Median time of diagnosis of diabetes mellitus post transplantation was 3 years (IQR: 0–7 years). The cohort with newly diagnosed diabetes mellitus exhibited a higher median age at transplantation (60 (54–63) versus 48 (42–57), *p*: 0.023) and a greater median BMI compared to the cohort without diabetes (34.1 (IQR: 29.4–37.2) versus 27.7 (IQR: 24.9–30.9), *p*: 0.004).

Hypertension was identified in 47 (21.1%) of the donors. Twenty-six individuals developed hypertension after transplantation. Median time from transplantation to hypertension diagnosis was 5.5 years (IQR: 2.75–8.25 years). The prevalence of eGFR < 60 mL/min/1.73 m^2^ was greater in hypertensive patients (34.7% vs. 20.8%, *p*: 0.042). Diabetes mellitus is a significant predictor of newly onset hypertension, correlating with an almost sevenfold increase in risk. This relationship remained statistically significant after adjusting for other variables in the multivariable model (OR: 6.73; 95% CI: 1.21–37.40; *p* = 0.029).

Ten donors (4.3%) encountered significant cardiac adverse events post-transplantation, with a median duration of 6.5 years (IQR: 4–7.25 years). Seven patients (3%) experienced a significant cardiac event prior to transplantation. The average age was greater for patients who experienced cardiac events (56.2 ± 9.1 vs. 48.75 ± 10.6, *p*: 0.001). The ASCVD group exhibited a significantly elevated probability of hypertension (*p* < 0.001). In the assessment of ASCVD and its associated risk factors, advanced age at transplantation (OR: 4.95; 95% CI: 1.02–23.89; *p* = 0.046) and male gender (OR: 4.09; 95% CI: 1.03–16.30; *p* = 0.045) were significantly correlated with an elevated risk of ASCVD in univariate analysis. However, no independent predictors were identified in the multivariable analysis.

The cumulative follow-up duration amounted to 1538 patient-years, involving 232 donors with a median follow-up period of 6 years (range 4–9 years). Four deaths were reported during the whole follow-up period. Median time to death from the time of transplantation was 6 years (IQR: 5.25–6.25 years). No cause of death was associated with cardiovascular disease. None of the deaths were attributable to donation. The causes of death were glioblastoma multiforme, lung cancer, pancreatic cancer, and postoperative complications following a cardiac ablation procedure.

## 4. Discussion

Donor nephrectomy inherently results in a decrease in nephron mass, a recognized risk factor for the onset of CKD [7]. Therefore, long-term surveillance of both kidney and cardiovascular health in kidney donors remains essential. This study, which evaluated the cardiac and kidney outcomes of living kidney donors, identified a remarkably low prevalence of ASCVD and diabetes but a relatively high incidence of decreased eGFR during follow-up. Significantly, age exceeding 50 years at the time of transplantation, a diminished pre-transplant eGFR, and the emergence of newly onset proteinuria during follow-up were found as potential risk factors for GFR decline. A recent single-center study in Türkiye identified male sex and a lower pretransplant GFR as independent risk factors, corroborating our findings [8].

In our study, 18.9% of patients exhibited a GFR below 60 mL/min/1.73 m^2^. The 2011 CREDIT study conducted in Türkiye revealed that the prevalence of stage 3 CKD was 4.7%, stage 4 CKD was 0.3%, and stage 5 CKD was 0.2% [9]. The observed rate of decline in eGFR below 60 mL/min/1.73 m^2^ in our donor group appears to exceed that of the general population. Research indicates that donor nephrectomy increases the long-term risk of ESKD [10,11]. Aside from donor nephrectomy, additional factors are believed to contribute to the increased risk of ESKD. Since most kidney donors provide their kidneys to relatives, a familial history of kidney disease may exaggerate the perceived risk [11]. In comparison to prior studies, the risk of ESKD may be diminished in non-relative transplant recipients lacking a familial history [12]. Among this study’s cohort, parents constituted the primary donor group (88 donors, 37.9%), followed by siblings (52 donors, 22.4%) and second-degree relatives (16 donors, 6.9%). Spousal donations constituted a significant percentage of cases (65 donors, or 28.0%), indicating the sociocultural endorsement of emotionally driven donations among family members. A 0.9% of donors were unrelated yet emotionally driven (*n* = 2), and 1.7% of donations (*n* = 4) transpired via cross-donation agreements. In total, over two-thirds of donations were from biologically related individuals, supporting that familial predispositions may influence post-donation renal functional patterns seen in certain donors. Challenges in engaging with the complete donor cohort hinder the ability to forecast outcomes. We successfully acquired current records for 232 (61.2%) of the 379 donors in our study within the designated time frame.

The research indicated that the ASCVD rate among the donors was 4.3%. The incidence of major adverse cardiac events was 1.7 events per 1000 patient-years in the donor group and 2 events in the control group, as reported in the study by Garg et al., which had a median follow-up of 6.5 years. The ASCVD levels of donors and the general population exhibited no significant differences [13]. A Norwegian cohort study involving 1901 donors and a median follow-up of 15.1 years indicated that the risk of ESKD was 11.38 times greater, cardiovascular mortality was 1.4 times greater, and all-cause mortality was 1.3 times greater compared to controls with similar characteristics. It was emphasized that following the tenth year, mortality curves began to diverge [10]. Cardiovascular mortality was similar to that of the control group in a follow-up study conducted in Korea, which monitored 1292 donors for an average duration of 12.3 ± 8.1 years, with a mean age of 40.7 ± 11.1 years. Within this cohort, merely 6 (0.36%) of the donors encountered a cardiac event [14].

In Türkiye, the prevalence of coronary heart disease was 6% among individuals aged 45–54, 17% among those aged 55–64, and 28% in the population over 65 years of age. These figures seem significantly elevated in comparison to European statistics [15]. Despite the seemingly low incidence of cardiovascular disease within our donor cohort of comparable age, it remains elevated in comparison to cohorts from Europe, the United States, and Korea. The diminished rate noted in the donor group compared to Turkish epidemiological studies’ reported rates may result from the stringent criteria employed prior to transplantation and the donors’ more rigorous compliance with lifestyle recommendations post-transplantation.

Our research indicates that 21.1% of the donor cohort exhibited hypertension. The TURDEP-II study in 2013 determined the incidence of hypertension in the community to be 31.4%, while the CREDIT study found it to be 32.7% [9,16]. Data indicates that the risk of hypertension does not increase during the initial stages. No significant alteration was observed in 24 h blood pressure monitoring during a prospective study with a three-year follow-up [17]. A 2006 meta-analysis of 48 studies involving 5145 donors revealed that, after 5 to 10 years of follow-up, the systolic blood pressure of donors increased by 5 mmHg more than that of the control group [18]. A study monitoring 1295 donors over a median duration of six years revealed that the donor cohort exhibited a 19% elevated incidence of post-transplant hypertension diagnoses [5]. The prevalence of hypertension was lower in our study than in the general population. However, long-term research indicates an approximately 20% increase in the risk of hypertension, although there is no discernible short-term risk increase [5,19].

The prevalence of DM was 3.4%, notably low in comparison to standard population rates. It is important to recognize that the development of new-onset diabetes after donation may reflect the donors’ underlying demographic and metabolic risk profiles rather than the donation procedure itself. Advanced age and increased BMI are recognized risk factors for type 2 diabetes, and these attributes likely influenced the incidence noted in our cohort. The TURDEP-II study revealed that 16.7% of individuals had diabetes mellitus [16]. Diabetes mellitus is a recognized risk factor for chronic kidney disease. It is the predominant etiology of chronic kidney disease globally. The etiology profile of ESKD in donors evolves over time, especially in the long term, with age-related conditions such as hypertension and diabetes mellitus becoming increasingly prevalent [20]. This study indicated that diabetes is an independent risk factor for the onset of hypertension; however, it failed to establish a heightened risk of ASCVD and GFR decline in diabetic donors, possibly due to the comparatively low prevalence of diabetes in the donor cohort.

Our research indicated that 32.2% of the donors were classified as obese after transplantation. The TURDEP-II study found that 35% of general population were obese, 44% of women, and 27% of men. The prevalence of obesity is swiftly rising in Turkey and is an established risk factor for diabetes mellitus, hypertension, and atherosclerotic cardiovascular disease. Our study’s obesity rate is comparable to that of the TURDEP-II study, and it is higher than that of the 2022 TÜİK health survey [16,21]. At our center, donor candidates with an elevated BMI undergo assessment to ensure metabolic safety before donation. Donation is allowed for individuals with a BMI of up to 35 kg/m^2^, provided that metabolic screening, including fasting glucose, lipid profile, blood pressure monitoring, and assessment for insulin resistance, reveals no abnormalities. Candidates are encouraged to adopt lifestyle modifications and weight optimization strategies; however, mandatory weight reduction is required only for individuals with a BMI ≥ 33 kg/m^2^ or those demonstrating metabolic derangements. This approach aims to balance donor safety with the practical realities and demographic characteristics of our donor population, in whom overweight status is relatively common.

This study investigates an extensive cohort of living kidney donors from a singular tertiary transplant center in Türkiye, where living donations constitute the predominant method of kidney transplantation. Our findings provide clinicians real-world data by presenting a comprehensive overview of donor health over a median duration of six years, encompassing cardiovascular risk and kidney health. This study emphasizes the necessity of formulating follow-up plans specific to regions with significant living donor populations, alongside enhancing the global evidence.

This retrospective study fills a significant gap in the existing literature by offering long-term outcome data on living kidney donors from a region where such evidence is notably scarce. Our cohort constitutes one of the most extensive single-center donor follow-up datasets in our country, providing a thorough assessment of kidney outcomes as well as metabolic and cardiovascular trajectories. The donor demographic in our study, marked by elevated obesity and hypertension rates relative to standard Western populations, mirrors the actual donor demographics found in numerous middle-income healthcare systems, thereby augmenting the applicability of our findings for transplant programs in analogous environments.

A further strength of this study is the identification and characterization of donors who exhibited post-donation eGFR < 60 mL/min/1.73 m^2^, a clinically significant subgroup that may enhance future donor risk stratification. Such detailed analyses are rarely found in regional reports and provide unique insights into donor variability.

This study should be regarded as a foundational, pioneering contribution that highlights the necessity for prospective controlled studies and multicenter collaborations to yield higher-level evidence and enhance donor selection and follow-up strategies.

However, it is essential to acknowledge several limitations. The study’s retrospective and single-center design may restrict generalizability. Establishing a direct correlation between observed outcomes and the act of donation is difficult in the absence of a corresponding non-donor control group. Moreover, the number of donors who were lost to follow-up may lead to selection bias. The limited number of events for specific outcomes, such as diabetes and ASCVD, may diminish the statistical power of certain outcome analyses.

Despite these constraints, this study collects data from actual scenarios to provide significant insights into the long-term consequences for living kidney donors. The primary advantages include the extensive sample size and prolonged follow-up duration, enabling a comprehensive assessment of alterations in cardiovascular risk and kidney function.

## 5. Conclusions

This study provides a thorough assessment of the long-term outcomes for living kidney donors in a real-world context. Despite low mortality rates, our findings implies that donors have substantial risks in some aspects. Even with the meticulous selection of donors, later evaluations revealed significant problems such as obesity, newly onset hypertension, and a decline in kidney functions.

Cardiovascular events were notably rare, and none of the donors experienced end-stage kidney disease. A decline in GFR below 60 mL/min/1.73 m^2^ is more common than in the general population compared to the historical cohorts, highlighting the need for lifestyle counseling and ongoing monitoring.

Our findings emphasize the importance of providing continuous care for donors even after surgery, despite the general safety of living donations. Donor health can be promoted and outcomes can be optimized following donation through suitable follow-up and risk-based approaches.

## Figures and Tables

**Table 1:** Demographic and Clinical Data of Donor Population, According to Sex.

	Female (*n* = 144)	Male (*n* = 88)	*p* Value	Total (*n* = 232)
At transplantation				
Age	48.5 ± 10.3	50.63 ± 10.99	0.139 ^a^	49.30 ± 10.46
Active smoking	16 (11%)	36 (40%)	**<0.001** ^b^	52 (22%)
Hypertension	14 (9.7%)	7 (7.9%)	0.649 ^b^	21 (9.1%)
ASCVD	4 (2.8%)	3 (3.4%)	0.785 ^b^	7 (3.01%)
eGFR (CKD-EPI, mL/min/1.73 m^2^)	104.87 ± 12.88	102.29 ± 12.30	0.134 ^a^	103.89 ± 12.70
CKD Stage				
G1	128 (88.9%)	77 (87.5%)		205 (88.3%)
G2	16 (11.1%)	11 (12.5%)		27 (11.7%)
G3				
G4				
G5				
UPCR (mg/g creatinine) (*n*:193)	89.40 ± 57.78	72.21 ± 33.64	0.01 ^a^	82.81 ± 50.52
Post-transplantation				
First week post-transplant eGFR (CKD-EPI, mL/min/1.73 m^2^)	70 (48, 130, 20)	65 (36, 114, 17)	**0.008** ^a^	68 (36, 130, 20)
3-month post-transplant (*n*:103) eGFR (CKD-EPI, mL/min/1.73 m^2^)	75.8 (43.3, 115.4, 20.3) (*n*:64)	68.5 (43.7, 118.1, 23.6) (*n*:39)	0.128 ^a^	73 (43.3, 118.1, 17.5)
First year post-transplant	70.5 (43, 113, 17)	66 (35, 102, 17)	0.459 ^a^	70 (35, 113, 17)
eGFR < 60 mL/min/1.73 m^2^ post-transplant	23 (16.1%)	21 (23.9%)	0.07 ^b^	44 (18.9%)
CKD Stage				
G1	19 (13.1%)	15 (17%)		34 (14.6%)
G2	102 (70.8%)	52 (59.1%)		154 (66.4%)
G3	23 (16.1%)	19 (21.6%)		42 (18.1%)
G4		2 (2.3%)		2 (0.9%)
G5				
Proteinuria (UPCR > 150 mg/g) (*n*:153)	16 (11.1%)	4 (4.5%)	0.096 ^b^	20 (8.6%)
Last UCPR (mg/g creatinine) *n*:153	111.15 ± 84.21	104.91 ± 49.31	0.611 ^a^	108.82 ± 73.05
BMI (kg/m^2^, *n* = 199)	29.32 ± 5.03	26.88 ± 4.06	**<0.001** ^a^	28.35 ± 4.81
Obesity (*n*:199)	47 (32.6%)	17 (19.3%)	**0.009** ^b^	64 (32.2%)
Newly onset ASCVD	3 (2.1%)	7 (7.9%)	**0.045** ^b^	10 (4.3%)
Newly onset HT	16 (11.1%)	10 (11.3%)	0.953 ^b^	26 (11.2%)
Newly onset Diabetes	6 (4.2%)	1 (1.1%)	0.258 ^b^	7 (3.01%)
SBP last follow-up	120 (90, 160, 18.75)	120 (100, 171, 16.25)	0.160 ^a^	120 (90, 171, 20)
DBP last follow-up	75 (60, 100, 10)	80 (50, 109, 13.5)	**0.03** ^a^	76 (50, 109, 10.5)
Death	1 (0.7%)	3 (3.4%)	0.154 ^c^	4 (1.7%)

^a^ Student’s *t*-test, ^b^ Pearson Chi-squared test, ^c^ Fisher’s Exact test. ASCVD: Atherosclerotic cardiovascular disease, BMI: Body mass index, DBP: Diastolic blood pressure, DM: Diabetes mellitus, GFR: Glomerular filtration rate, SBP: Systolic blood pressure.

**Table 2:** Demographic and Clinical Data of Donor Population, according to eGFR below 60 mL/min/1.73 m^2^.

	eGFR < 60 mL/min/1.73 m^2^ (*n* = 44)	eGFR ≥ 60 mL/min/1.73 m^2^ (*n* = 188)	*p* Value	Total (*n* = 232)
At transplantation				
Age	54.72 ± 10.09	48.03 ± 10.39	**<0.001** ^a^	49.30 ± 10.46
Sex (Female)	23 (52.3%)	121 (64.4%)	0.137 ^b^	144 (62.1%)
Active smoking	12 (27.3%)	40 (21.3%)	0.391 ^b^	52 (22.4%)
Hypertension	7 (15.9%)	14 (7.4%)	0.086 ^c^	21 (9.1%)
ASCVD	3 (6.8%)	4 (2.1%)	0.128 ^c^	7 (3.0%)
eGFR (CKD-EPI, mL/min/1.73 m^2^)	92.79 ± 11.48	106.5 ± 11.53	**<0.001** ^a^	103.89 ± 12.70
UPCR (mg/g creatinine) (*n*:193)	84.10 ± 63.01	82.47 ± 46.95	0.880 ^a^	82.81 ± 50.52
Post-transplantation				
First week post-transplant eGFR (CKD-EPI, mL/min/1.73 m^2^)	57.5 (36, 80, 10.25)	71 (48, 130, 18)	**<0.001** ^a^	68 (36, 130, 20)
3-month post-transplant (*n*:103) eGFR (CKD-EPI, mL/min/1.73 m^2^)	56.4 (43.3, 77.2, 14.3) (*n*:17)	75.8 (43.7, 118.1, 19.2) (*n*:86)	**<0.001** ^a^	72.98 (43.3, 118.1, 17.5)
First year post-transplant eGFR (CKD-EPI, mL/min/1.73 m^2^)	60.5 (42, 78, 17.75)	72 (35, 113, 16)	**<0.001** ^a^	70 (35, 113, 17)
Proteinuria (UPCR > 150 mg/g)	9 (20.5%)	11 (5.9%)	**0.005** ^c^	20 (8.6%)
Last UPCR (mg/g creatinine) (*n*:141)	145.72 ± 129.52 (*n*:31)	100.78 ± 46.81 (*n*:110)	0.066 ^a^	110.67 ± 75.16
BMI (kg/m^2^, *n* = 199)	27.85 ± 4.97	28.48 ± 4.78	0.465 ^a^	28.35 ± 4.81
Obesity	11 (27.5%)	53 (33.3%)	0.48 ^b^	64 (32.2%)
Newly onset ASCVD	4 (9.1%)	6 (3.2%)	0.099 ^c^	10 (4.3%)
Newly onset HT	6 (13.6%)	20 (10.6%)	0.597 ^c^	26 (11.2%)
Newly onset Diabetes	3 (6.8%)	4 (2.1%)	0.128 ^c^	7 (3.01%)
SBP last follow-up	120 (102, 171, 20)	120 (90, 170, 17)	0.286 ^a^	120 (90, 171, 20)
DBP last follow-up	80 (60, 105, 20)	75 (50, 109, 10)	0.206 ^a^	76 (50, 109, 10.5)
Death	2 (4.5%)	2 (1.1%)	0.164 ^c^	4 (1.7%)

^a^ Student’s *t*-test, ^b^ Pearson Chi-squared test, ^c^ Fisher’s Exact test. ASCVD: Atherosclerotic cardiovascular disease, BMI: Body mass index, DBP: Diastolic blood pressure, DM: Diabetes mellitus, GFR: Glomerular filtration rate, SBP: Systolic blood pressure, CKD: chronic kidney disease.

**Table 3:** Logistic regression analysis of eGFR below 60 mL/min/1.73 m^2^) and related risk factors in living kidney donors (LKDs).

	Univariate		Multivariate (Adjusted)	
	OR (95% Cl)	*p*-Value ^d^	OR (95% Cl)	*p*-Value ^d^
Sex	0.61 (0.31–1.18)	0.139	0.55 (0.24–1.24)	0.147
Age at transplantation > 50	3.68 (1.78–7.59)	<0.001	1.27 (0.52–3.13)	0.605
Pre-Transplantation eGFR	0.91 (0.88–0.94)	<0.001	0.91 (0.88–0.95)	<0.001
ASCVD	3.37 (1.20–9.42)	0.021	2.63 (0.72–9.64)	0.143
Proteinuria	4.14 (1.60–10.73)	0.003	6.61 (1.98–22.07)	0.002
DM	0.30 (0.06–1.38)	0.121	3.68 (0.60–22.78)	0.161
HT	1.90 (0.90–4.01)	0.092	2.17 (0.85–5.53)	0.106
Obesity	0.76 (0.35–1.63)	0.481		
Smoking	0.72 (0.34–1.53)	0.392		

d. Logistic regression analysis. ASCVD: atherosclerotic cardiovascular disease, eGFR: estimated glomerular filtration rate, DM: diabetes mellitus, HT: hypertension.

## Data Availability

The datasets generated and analyzed during the current study are available from the corresponding author upon reasonable request.

## Abbreviations

The following abbreviations are used in this manuscript:

ASCVD	Atherosclerotic Cardiovascular disease
BMI	Body Mass Index
CKD	Chronic Kidney Disease
DBP	Diastolic Blood Pressure
DM	Diabetes Mellitus
eGFR	Estimated Glomerular Filtration Rate
ESKD	End-stage Kidney Disease
HT	Hypertension
IQR	Interquartile range
OR	Odds Ratio
SBP	Systolic Blood Pressure
UPCR	Urine protein-to-creatinine ratio
